# Modulation of Contact Resistance of Dual‐Gated MoS_2_ FETs Using Fermi‐Level Pinning‐Free Antimony Semi‐Metal Contacts

**DOI:** 10.1002/advs.202301400

**Published:** 2023-05-05

**Authors:** Tien Dat Ngo, Tuyen Huynh, Hanggyo Jung, Fida Ali, Jongwook Jeon, Min Sup Choi, Won Jong Yoo

**Affiliations:** ^1^ SKKU Advanced Institute of Nano Technology Sungkyunkwan University Suwon Gyeonggi‐do 16419 Republic of Korea; ^2^ Department of Electrical and Electronics Engineering Konkuk University Seoul 05029 Republic of Korea; ^3^ Department of Electronics and Nanoengineering Aalto University P.O. Box 13500 Espoo FI‐00076 Finland; ^4^ Department of Materials Science and Engineering Chungnam National University Daejeon 34134 Republic of Korea

**Keywords:** 2D semiconductors, contact resistance, dual‐gate, Fermi level pinning‐free, four‐point‐probe measurements, junction resistance, semimetal

## Abstract

Achieving low contact resistance (*R*_C_) is one of the major challenges in producing 2D FETs for future CMOS technology applications. In this work, the electrical characteristics for semimetal (Sb) and normal metal (Ti) contacted MoS_2_ devices are systematically analyzed as a function of top and bottom gate‐voltages (*V*_TG_ and *V*_BG_). The semimetal contacts not only significantly reduce *R*_C_ but also induce a strong dependence of *R*_C_ on *V*_TG_, in sharp contrast to Ti contacts that only modulate *R*_C_ by varying *V*_BG_. The anomalous behavior is attributed to the strongly modulated pseudo‐junction resistance (*R*_jun_) by *V*_TG_, resulting from weak Fermi level pinning (FLP) of Sb contacts. In contrast, the resistances under both metallic contacts remain unchanged by *V*_TG_ as metal screens the electric field from the applied *V*_TG_. Technology computer aided design simulations further confirm the contribution of *V*_TG_ to *R*_jun_, which improves overall *R*_C_ of Sb‐contacted MoS_2_ devices. Consequently, the Sb contact has a distinctive merit in dual‐gated (DG) device structure, as it greatly reduces *R*_C_ and enables effective gate control by both *V*_BG_ and *V*_TG_. The results offer new insight into the development of DG 2D FETs with enhanced contact properties realized by using semimetals.

## Graphical abstract

Dual‐gated MoS_2_ field‐effect transistors are fabricated using semimetal (antimony, Sb) as top surface electrodes and hexagonal boron nitride as a top‐gate (TG) dielectric. This approach achieves Fermi level pinning (FLP)‐free contacts, which enable a strong modulation of pseudo‐junction resistance by TG voltages, owing to the weak FLP.

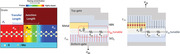

## 1 Introduction

Transition‐metal dichalcogenides (TMDs) are a promising candidate for semiconducting channel materials that are expected to be useful in future CMOS devices due to their bodies that are atomically thin and free of dangling bonds, thus enabling efficient electrostatic control at very low voltages. One of the most studied materials among this TMD family is MoS_2_. In recent years, there have been many efforts aiming to further improve the performance of 2D‐based FETs, e.g., contact engineering.^[^ ^1^, ^2^, ^3^, ^4^, ^5^, ^6^, ^7^ ^]^ However, the notoriously high contact resistance (*R*_C_) in MoS_2_ FETs hinders the realization of high‐performance electronic devices. Several techniques have recently been reported to improve *R*_C_ by utilizing semimetals as contact metals for MoS_2_ FETs, which can substantially suppress the metal‐induced gap states (MIGS).^[^ ^8^, ^9^, ^10^, ^11^, ^12^, ^13^, ^14^, ^15^ ^]^

Nonetheless, most studies have investigated the contact properties in a single‐gated (SG) architecture rather than a dual‐gated (DG) architecture. Conventional SG planar devices encounter a severe challenge such as poor electrostatic controllability due to their short channel effects, which hinders further scaling of the devices. By contrast, the DG can enable more efficient control of the channel resistance of 2D layered material‐based FET devices, and it can also be used for high‐performance and variously designed gate‐integrated CMOS circuits.^[^ ^16^, ^17^, ^18^, ^19^, ^20^ ^]^ Several studies have used multi‐bridge channel FETs, which are analogous to a DG structure, to achieve excellent electrostatic controllability and power/area efficiency.^[^ ^21^, ^22^, ^23^, ^24^ ^]^ Moreover, a research group has recently explored DG MoS_2_ FETs with a channel length of 25 nm, which show a comparable performance to state‐of‐the‐art Si technology.^[^ ^25^ ^]^ Thus, the DG architectures of high‐performance 2D FETs need to be developed to ensure a long‐term continuity of Moore's Law scaling.

Meanwhile, some studies have shown that overall *R*_C_ is independent of top‐gate voltage (*V*_TG_) in DG FETs, because the *V*_TG_‐induced electric field can be screened by metal electrodes, resulting in unchanged intrinsic resistance under the contacts (*R*_ci_).^[^ ^19^, ^20^ ^]^ It should be noted that the normal metals used in these studies induce strong Fermi level pinning (FLP) at the metal–semiconductor (MS) interface; therefore, the lateral junction resistances near the contacts are also unchanged by *V*_TG_.^[^ ^26^, ^27^, ^28^ ^]^ Recently, to reveal such FLP‐dependent contact behaviors, the classical model on contact interface between metal and semiconductor involving specific contact resistivity (*ρ*_c_) and sheet resistance under the metal contact (*R*_sk_) has been further developed by adding pseudo‐lateral junction resistance (*R*_jun_) components to the total *R*_C_ in 2D‐based FETs.^[^ ^29^, ^30^, ^31^ ^]^ Venica et al. first proposed the use of the *R*_jun_ component to explain the gate‐tunable *R*_C._^[^ ^30^ ^]^ Moreover, Ber et al. proposed a unique approach to specifically probe the *R*_jun_ component by 4‐point probe (4PP) measurements, which also helps to extract various metallic contact‐related resistance components.^[^ ^29^ ^]^ Meanwhile, Kong et al. performed a DFT band calculation that showed clear differences in the contact resistance components between FLP‐dominant and FLP‐free metal contact platforms.^[^ ^32^ ^]^

In this work, we used a semimetal (antimony, Sb) as a top surface electrode and hBN as a top‐gate (TG) dielectric for the purpose of realizing an FLP‐free MS interface in DG MoS_2_ FETs. Ohmic contact is realized with the Sb contact by suppressing MIGS at the Sb and MoS_2_ interface.^[^ ^8^, ^9^ ^]^ We performed systematic 4PP measurements to extract *R*_C_ as a function of bottom‐gate voltage (*V*_BG_) and *V*_TG_. Interestingly, our Sb‐contacted devices showed that *R*_C_ is modulated sensitively by both *V*_TG_ and *V*_BG_, which is in sharp contrast with Ti‐contacted devices showing *R*_C_ modulation only by *V*_BG_. To further understand this anomalous contact behavior of Sb‐contacted devices, we extracted *R*_jun_ and *R*_ci_ as functions of DG voltages.^[^ ^29^ ^]^ The results showed that *R*_jun_ is significantly modulated by both *V*_TG_ and *V*_BG_ while *R*_ci_ is modulated only by *V*_BG_. By contrast, *R*_jun_ and *R*_ci_ of Ti‐contacted devices are weakly tuned by *V*_TG_ due to strong FLP_._ This work suggests that the FLP‐free semimetal contacts can be used to efficiently modulate *R*_C_ in DG MoS_2_ FETs by applying both *V*_BG_ and *V*_TG_. Technology computer‐aided design (TCAD) simulations further confirm the role of *V*_TG_ in improving *R*_jun_ toward high‐performance Sb‐contacted DG MoS_2_ devices.

## 2 Results and Discussion

**Figure 1:**
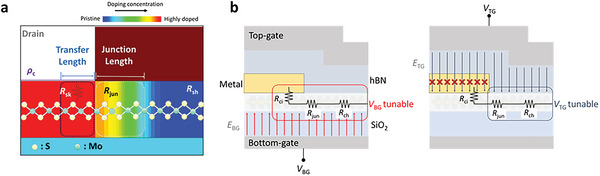
a) Schematic of a MoS_2_ FET at the drain contact side showing doping concentration profile at the contact and channel regions. b) Schematic of a DG MoS_2_ FET showing *R*_C_ components that consist of *R*_ci_ and *R*_jun_. The *E*_TG_ is screened by the metal electrode, while the *E*_BG_ can tune all the *R*_C_ components.

**Figure** **1**a shows a schematic of MoS_2_ FET with *R*_C_ components at the MS contact. To systematically investigate the impacts of *V*_BG_ and *V*_TG_ on overall *R*_C_, we decompose *R*_C_ into *R*_ci_ and *R*_jun_.^[^ ^29^, ^30^, ^31^ ^]^ *R*_ci_ is the resistance component under the electrode that consists of *ρ*_c_ and *R*_sk_. Here, when using our FLP‐free devices, the gate‐tunable *R*_jun_ is mostly attributable to the difference in doping concentration between MoS_2_ under the contact and at the channel area that originates from heavy n‐type doping in MoS_2_ by semimetal Sb, forming a gate‐tunable n^+^‐n junction. Figure 1b represents a schematic of a DG MoS_2_ FET showing the impact of *V*_TG_ and *V*_BG_ on contact resistance components of the device. As Ber et al. reported, *R*_ci_ is weakly dependent on *V*_BG_ due to FLP and high doping concentration under the contact.^[^ ^29^ ^]^ Moreover, *R*_ci_ is supposed to be independent of *V*_TG_, as the electric field induced by *V*_TG_ (*E*_TG_ as indicated in the diagram) is screened by the covered metal electrodes. In contrast, *R*_jun_ is found to be *V*_BG_ tunable due to the modulation of lateral junction potential.^[^ ^29^, ^30^, ^31^ ^]^ Furthermore, *R*_jun_ is supposed to be strongly modulated by *V*_TG_ unlike *R*_ci_ as it is not covered by the metal electrode.^[^ ^29^, ^30^, ^31^ ^]^ Thus, we believe that overall *R*_C_ could be affected by applying *V*_BG_ and *V*_TG_ simultaneously in the DG structure considering such strong modulation of *R*_jun_.

To investigate the impact of *V*_TG_ on *R*_C_, we fabricated DG MoS_2_ FETs with Sb (and DG MoS_2_ FETs with Ti as a reference) electrodes on a global bottom‐gate (BG) silicon substrate capped with 285 nm SiO_2_ (To ensure the screening effect of metal electrode against *V*_TG_, total thickness of ≈25 nm is used).^[^ ^33^ ^]^ The TG of the device is formed by an exfoliated hBN flake (≈50 nm) with a TG electrode of Ti/Au (5/70 nm). The detailed fabrication process is described in the Experimental Section. The Raman spectra and atomic force microscopic (AFM) images of MoS_2_ and hBN are shown in Figure S1 (Supporting Information). The dry‐transferred hBN possibly results in the formation of air gap at the metal‐MoS_2_ junction where the top hBN is not fully contacted with MoS_2_ channel. However, if we consider the air gap as a dielectric layer with a dielectric constant of 1, MoS_2_ area at the gap is still electrostatically tunable by *V*_TG_. In this sense, Zhang et al. have reported that 2D semiconductors can be electrostatically doped by applying gate‐voltages despite the existence of air gap at the metal‐2D semiconductor junction.^[^ ^33^ ^]^ Therefore, although the existence of the air gap at metal‐MoS_2_ junction may affect the top‐gate performance, the electrostatic modulation of *R*_C_ and *R*_jun_ by *V*_TG_ is still possible.

**Figure 2:**
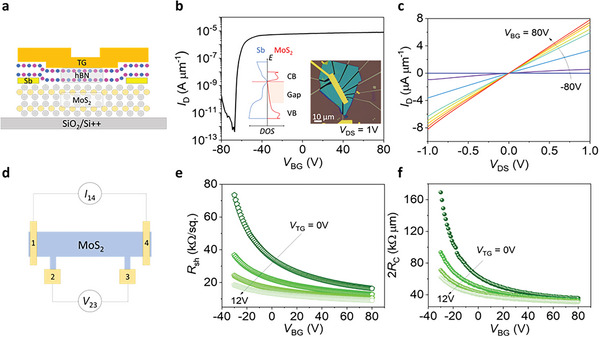
a) Schematic of a Sb‐contacted DG MoS_2_ FET. b) Transfer characteristics of the FET with *V*_DS_ = 1 V and *V*_TG_ = 0 V. The insets show an optical image of the device and a band structure showing the DOS of Sb and MoS_2_. c) Output characteristic of the FET by varying *V*_BG_ = −80 to 80 V (*V*_TG_ = 0 V). d) Schematic of a 4PP measurement configuration of the FET. e) *R*_sh_ and f) 2*R*_C_ as a function of *V*_BG_ at different *V*_TG_ obtained from the FET.

**Figure 3:**
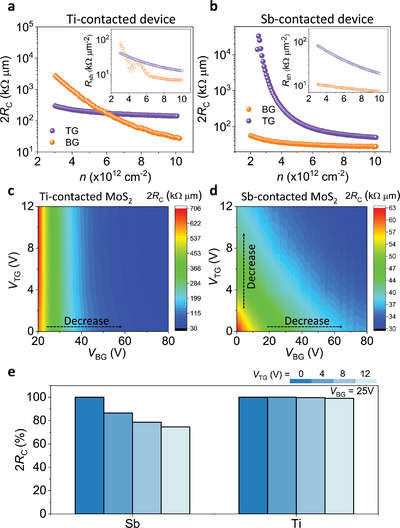
2*R*_C_ of a) Ti‐ and b) Sb‐contacted devices measured by 4PP measurements with separate TG and BG biases. The inset shows *R*_sh_ as a function of carrier densities. 2*R*_C_ map as a function of *V*_TG_ and *V*_BG_ for c) Ti‐ and d) Sb‐contacted DG MoS_2_ FETs. e) Modulated percentages of 2*R*_C_ as a function of *V*_TG_ at a fixed *V*_BG_ = 25 V.

**Figure** **2**a illustrates the final structure of the DG MoS_2_ FET. The transfer characteristic of the DG FET with Sb contacts is depicted in Figure 2b. The device with *L*_CH_ = 11.5 µm achieves *I*_ON_ of ≈8 µA µm^−1^ at *V*_DS_ = 1 V with a good *I*_ON_ */I*_OFF_ ratio of ≈10^7^ without *V*_TG_. The Ohmic behavior of Sb contact is evidenced by the linear output characteristic, as represented in Figure 2c. It is noted that the linear behavior is also obtained without gating the contact area by sweeping *V*_TG_ from −12 to 12 V at *V*_BG_ = 0 V (Figure S2, Supporting Information), which shows the superiority of Sb semimetal over normal metals. The mobilities and hysteresis behavior of Sb‐contacted DG MoS_2_ FETs extracted from the transfer curves with *V*_TG_ and *V*_BG_ are shown in Figures S3 and S4 (Supporting Information). As a reference, the transfer and output characteristics of Ti‐contacted DG MoS_2_ FET are shown in Figure S5 (Supporting Information). Unlike Sb contacts, asymmetric output characteristics are observed with both *V*_TG_ and *V*_BG_ in Ti‐contacted device. We further demonstrate the distinctive *V*_TG_‐dependent transfer characteristics of the Sb‐contacted device compared to the Ti‐contacted device as shown in Figure S6 (Supporting Information). Both devices show a similar improvement in on‐state current when *V*_TG_ increases; however, the threshold voltage (*V*_th_) of the Sb‐contacted device is strongly shifted to negative *V*_TG_, while the Ti‐contacted device revealed a slight *V*_th_ shift. The origin of this phenomenon can be explained by the different *V*_TG_‐dependent *R*_C_ between both devices as discussed in **Figure** **3**.

To evaluate the FLP behavior of the devices, we further conducted temperature‐dependent measurements of the transfer characteristics to extract Schottky barrier height (SBH), as depicted in Figure S7 (Supporting Information). The Sb‐contacted device shows a negligible SBH (≈0 meV), while the Ti‐contacted device shows a relatively high SBH (≈200 meV) despite the fact that both the metals have similar work functions (4.4 eV for Sb and 4.33 eV for Ti).^[^ ^8^, ^34^ ^]^ The FLP behavior of both devices can be understood by band alignment, as depicted in Figure S7d,e (Supporting Information). The negligible SBH of the Sb‐contacted device is consistent with intrinsic band alignment showing FLP‐free contact for the 2D MoS_2_ FET. This FLP‐free behavior is consistent with the DFT calculation performed by Chou et al., which shows suppressed MIGS (inset of Figure 2b).^[^ ^9^ ^]^ Ti is supposed to have Ohmic contact with 2D MoS_2_ (≈0 meV) according to the isolated band structure, but the actual SBH is ≈200 meV, implying a strong FLP.

Figure 2d illustrates the device configuration used in the 4PP measurements to extract the *R*_C_ of the DG MoS_2_ devices. A fixed current (*I*_14_ = 0.33 µA·µm^−1^) is applied between electrodes 1 and 4, and the voltage is probed at each electrode. By measuring the voltage drop between electrodes 2 and 3 (Δ*V*_23_), sheet resistance (*R*_sh_) can be calculated by:

(1) $$R_{sh}=\frac{\Delta V_{23}}{I_{14}\times L_{23}}$$

Moreover, *R*_C_ can be calculated by:

(2) $$2R_{C}=\frac{\Delta V_{14}}{I_{14}}-\frac{\Delta V_{23}\times L_{14}}{I_{14}\times L_{23}}$$

where *L*_14_ = 11.5 µm and *L*_23_ = 5.8 µm are the distances between electrodes 1–4 and 2–3, respectively. Figure 2e exhibits *R*_sh_ as a function of *V*_BG_ that shows strong gate‐tunability with *V*_BG_ and *V*_TG_, since *R*_ch_ is gate‐tunable, as depicted in Figure 1b. We observed a noticeable reduction in *R*_sh_ from 16 to 9 kΩ per square when *V*_TG_ increases from 0 to 12 V, which is a typical behavior for n‐type semiconductors. This observation is consistent with previous results for DG 2D FETs.^[^ ^17^, ^19^, ^20^ ^]^ Figure 2f presents the *R*_C_ as a function of *V*_TG_ derived from Equation (2). Interestingly, we noticed a considerable improvement in *R*_C_ (63 to 40 kΩ µm at *V*_BG_ = 0 V) with the increase in *V*_TG_ from 0 to 12 V. This behavior can be reproduced in other separately prepared devices as shown in Figure S8 and S9. In all devices, *R*_C_ of Sb‐contacted device strongly depends on *V*_TG_. The strong *V*_TG_‐tunability of overall *R*_C_ in Sb‐contacted DG MoS_2_ FETs is in contrast to the results obtained from the previous studies with normal metal contacts.^[^ ^19^, ^20^ ^]^ The almost unchanged *R*_C_ regardless of *V*_TG_ in these studies can be attributed to the screening effect of *E*_TG_ by top‐contact metals and strong FLP.^[^ ^19^, ^20^ ^]^

For a comparison, we also performed the same 4PP measurement with Ti‐contacted DG MoS_2_ FETs. The *R*_sh_ and *R*_C_ values measured at different *V*_TG_ and *V*_BG_ of the devices are presented in Figure S10 (Supporting Information). The 2*R*_C_ and *R*_sh_ measured with separate *V*_TG_ and *V*_BG_ for both the Sb‐ and Ti‐contacted devices are represented in Figure 3a,b. For a fair comparison, we plotted the *R*_sh_ and *R*_C_ as a function of carrier concentration, which is extracted by the equation, *n* = *q* ^−1^ *C*_TG_(*V*_TG_ − *V*_th_), where *q* is the electron charge and *C*_TG_ is the TG capacitance ( = 1.23 × 10^−7^ F cm^−2^ for a 50 nm thick top hBN gate dielectric). The *R*_sh_ of both devices demonstrate a similar trend and values; however, *R*_C_ tunability is clearly different. The *R*_C_ of the Sb‐contacted device is strongly *V*_TG_ tunable, while a weak *V*_TG_‐dependent *R*_C_ is obtained with the Ti‐contacted device. Thus, we think that the difference in transfer characteristics of both the devices (different *V*_th_ shift with *V*_TG_ in Figure S6, Supporting Information) is mostly originated from the strong *V*_TG_‐dependent *R*_C_ of the Sb‐contacted device compared to the weak *V*_TG_‐dependent *R*_C_ of the Ti‐based device, as shown in Figure 3a,b.

Based on Figure 2f and Figure S10b (Supporting Information), we reproduced *R*_C_ map to visualize the different trends in *R*_C_ between Ti and Sb contacts depending on *V*_TG_ and *V*_BG_, as depicted in Figure 3c,d. For the Ti‐contacted device, *R*_C_ is modulated by *V*_BG_, while it remains unchanged by *V*_TG_, as shown in the color gradient of the map, indicating that *R*_C_ is dominated by *V*_BG_ (Figure 3a). This phenomenon has also been observed in previous studies with the same device architecture and normal metals.^[^ ^19^, ^20^ ^]^ By contrast, a distinctive behavior is observed with the Sb‐contacted device; *R*_C_ varies largely by both *V*_BG_ and *V*_TG_, as can be seen from the continuous variation of color gradient in Figure 3d. Figure 3e presents such 2*R*_C_ change in percentage as a function of *V*_TG_, showing a roughly 30% improvement when *V*_TG_ increases from 0 to 12 V with Sb contacts. However, Ti contact reduces 2*R*_C_ by merely ≈1% with the same *V*_TG_ condition.

**Figure 4:**
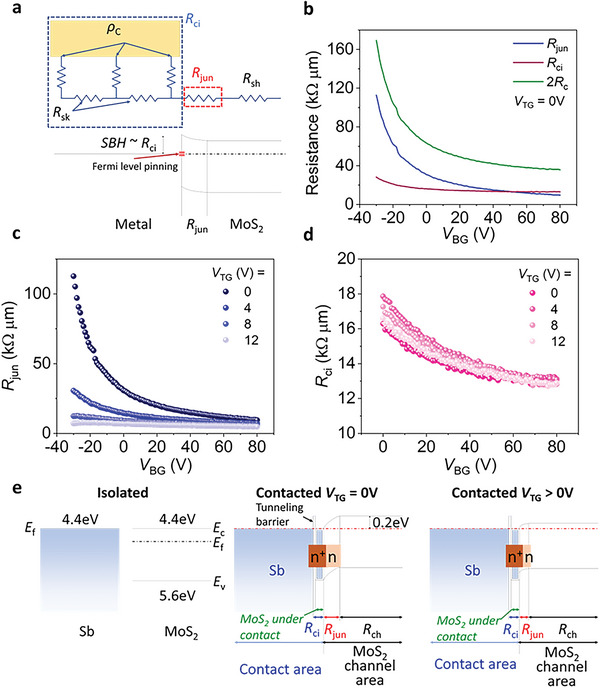
a) An equivalent circuit with resistance components at the MS contact and a corresponding band diagram illustrating FLP. b) *R*_ci_, *R*_jun_, and 2*R*_C_ as a function of *V*_BG_ at *V*_TG_ = 0 V, and c) *R*_jun_ and d) *R*_ci_ as a function of *V*_BG_ and *V*_TG_ for the Sb‐contacted DG MoS_2_ FET. e) Band diagrams for Sb‐contacted MoS_2_ illustrating how *R*_jun_ is modulated by *V*_TG_.

To elucidate the origin of the anomalous behavior of Sb‐contacted device, we utilized the method proposed by Ber et al.^[^ ^29^ ^]^ to extract *R*_jun_ and *R*_ci_ based on the 2*R*_C_ value obtained by 4PP measurements, thereby identifying predominant resistance components in *R*_C_. **Figure** **4**a illustrates an equivalent circuit and a corresponding band structure, including *R*_jun_ and *R*_ci_ at the MS interface.^[^ ^29^, ^30^ ^]^ The total contact resistance is expressed by:

(3) $$2R_{C}=2R_{ci}+R_{jun}$$

Here, *R*_ci_ is multiplied by 2 as the current passes through the source and drain electrodes. *R*_jun_ is found at the contact where the current is injected, thus, it is considered to have appeared only once in the above expression.^[^ ^29^ ^]^ Next, the resistance between the current injecting electrode and the nearest voltage probing electrode (*R*_12_) is expressed as:

(4) $$R_{12}=\frac{\Delta V_{12}}{I_{14}}=R_{ci}+R_{jun}+R_{sh}L_{12}$$

where *L*_12_ ( = 3.2 µm) is the distance between electrodes 1 and 2. Based on Equations (3) and (4), we extracted *R*_jun_ and *R*_ci_ using the value of 2*R*_C_ obtained through 4PP measurements. Figure 4b shows the calculated *R*_jun_ and *R*_ci_ values of the Sb‐contacted device at *V*_BG_ = ‐30 to 80 V and *V*_TG_ = 0 V. It should be noted that *R*_jun_ is strongly dependent on *V*_BG_, unlike *R*_ci_. *R*_jun_ can mainly originate from the strong electron doping effect of semimetal to MoS_2_ underneath the contact, thus resulting in the formation of n^+^ – n junction, where an n‐type region is located at the channel side.^[^ ^8^, ^29^ ^]^ The contribution of SBH can be negligible in *R*_jun_ due to the FLP‐free nature of semimetal contacts, as shown in Figure S7 (Supporting Information). Therefore, the potential barrier at the junction can be reduced when *V*_BG_ increases because the n‐region becomes a highly doped n^+^‐state as a result of electrostatic doping due to weak FLP. However, the n^+^‐region underneath the semimetal is not significantly affected by *V*_BG_ due to its high electron concentration.^[^ ^6^, ^35^, ^36^ ^]^

Figure 4c shows *R*_jun_ of the Sb‐contacted DG device as a function of *V*_BG_ with different *V*_TG_. Interestingly, *R*_jun_ is highly modulated by *V*_TG_, confirming that *E*_TG_ can modulate *R*_C_. At high *V*_TG_ = 12 V, *R*_jun_ becomes independent of *V*_BG_. This validates the fact that the electrostatic doping effect of *E*_TG_ significantly suppresses the potential barrier, equalizing the electron concentration of both MoS_2_ under the metal contact and at the channel area. In contrast, *R*_ci_ is very weakly dependent on *V*_TG_ as shown in Figure 4d, which can be understood as the metal screening effect. Thus, the improvement in *R*_C_ by *V*_TG_, as shown in Figure 2f and 3b, is mostly attributed to the reduced *R*_jun_.

The band diagram in Figure 4e describes the mechanism by which *V*_TG_ adjusts *R*_jun_. At *V*_TG_ = 0 V, the potential barrier is formed by band alignment between MoS_2_ and Sb based on their work functions. As a result, MoS_2_ underneath Sb contact is strongly n‐type doped, forming a n^+^‐n lateral junction with MoS_2_ at the channel area. Therefore, the n^+^‐n lateral junction dominantly contributes to *R*_jun_. When *V*_TG_ increases, the Fermi level of MoS_2_ at the channel area is expected to move upward, leading to a reduced potential barrier. Moreover, the depletion width in n^+^‐n lateral junction decreases by equalizing the doping concentration between MoS_2_ underneath the contact and at the channel area. This reduction in potential barrier and depletion width at high *V*_TG_ contributes to the decreased *R*_jun_.

For the Ti‐contacted device, the extracted *R*_jun_ and *R*_ci_ are presented in Figure S10c,d (Supporting Information). Unlike the Sb‐contacted device, *R*_jun_ is weakly dependent on *V*_TG_, presumably due to strong FLP. Since the Fermi level of MoS_2_ is strongly pinned at the MIGS or defect states, it barely shifts by *V*_TG_, particularly at the MS interface. Since *R*_ci_ is also independent of *V*_TG_, *R*_C_ for the Ti‐contacted device appears to be overall unchanged by *V*_TG_. This interpretation is further supported by a recent work on DFT calculation, which shows tunable band alignment of MS contact with Sb contacts due to their lack of MIGS, while unnoticeable tunability is observed with normal metals.^[^ ^13^ ^]^ Consequently, our work provides a distinctive advantage of semimetal‐contacted DG device in terms of its ability to modulate *R*_C_ with *V*_TG_.

**Figure 5:**
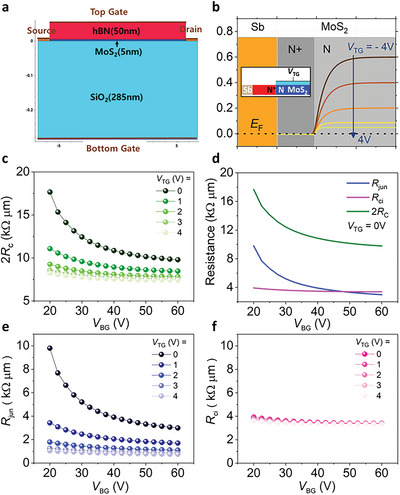
a) Device structure for TCAD simulations. b) Band alignment between Sb and MoS_2_ with varied *V*_TG_. c) Simulated 2*R*_C_ of Sb‐contacted DG device as a function of *V*_TG_ and *V*_BG_. d) Simulated *R*_ci_, *R*_jun_, and 2*R*_C_ as a function of *V*_BG_ at *V*_TG_ = 0 V. Simulated e) *R*_jun_ and f) *R*_ci_ as a function of *V*_BG_ and *V*_TG_.

Last, we verify our experimental results and mechanism by performing TCAD simulations. **Figure** **5**a shows a device structure of Sb‐contacted DG device used for our simulations (detailed information and parameters are described in the Experimental Section and Table S1, Supporting Information). The simulation assumes that the SBH of Sb‐contacted DG device to be zero due to its FLP‐free nature, as confirmed by our SBH measurement in Figure S7 (Supporting Information). Furthermore, the n^+^‐n junction is considered at the MS junction as Sb semimetal can induce heavy n‐type doping to MoS_2_. As shown in Figure 5b, the potential barrier of Sb‐contacted DG device significantly decreases as *V*_TG_ increases, which is consistent with our above postulation on strong modulation of *R*_C_ and *R*_jun_ by *V*_TG_. The strong modulation can also be seen in the simulation results from Figure 5c,e. Moreover, the simulated *R*_ci_ remains almost unchanged as depicted in Figure 5f due to the heavy n‐doping effect of Sb, which is also in good agreement with our experimental results. For the Ti‐contacted device, however, the simulated 2*R*_C_ remains constant with varied *V*_TG_ attributed to weak tunability of *R*_jun_ and *R*_ci_ (Figure S11), due to strong FLP. We performed further TCAD simulation to investigate how the transfer length (*L*_T_) and junction length (*L*_jun_) affect resistance components of the devices, as presented in Figures S12 and S13 (Supporting Information). When *L*_T_ increases up to 50 nm, *R*_jun_ of the device slightly decreases, while *R*_jun_ remains almost unchanged when *L*_T_ is greater than 50 nm. Thus, we believe that a further increase in *L*_T_ does not modulate the *R*_jun_ significantly, as depicted in Figure S12. Meanwhile, we found that increased *L*_jun_ results in a significant increase in *R*_jun_, while *R*_ci_ remains almost constant regardless of *L*_jun_, as shown in Figure S13 (Supporting Information). Here, *L*_jun_ is much more dominant in modulating *R*_jun_ than *L*_T_ when comparing Figures S12 and S13 (Supporting Information), suggesting *L*_jun_ is a key parameter for understanding the contact properties of 2D devices. Consequently, the TCAD simulations further demonstrate the origin of distinctive gate‐dependent contact parameters between Ti and Sb contacts.

## 3 Conclusion

In conclusion, we conducted a comprehensive study on the electrical properties of MoS_2_ devices contacted with semimetal (Sb) and normal metal (Ti) electrodes as a function of *V*_TG_ and *V*_BG_. The Sb‐contacted FETs revealed not only the negligible Schottky barrier but also a new insight on the role of DG structure to modulate *R*_C_, which has not been reported in previous reports. The DG structure combining with semimetal contacts revealed the strong dependence of *R*_C_ on *V*_TG_‐when compared to the devices with normal metallic contacts. We utilized a new model that considers *R*_jun_ component to the total *R*_C_, which shows that the weak FLP nature of semimetals modulates *R*_jun_ and is responsible for the significantly modulated *R*_C_ by *V*_TG_. In contrast, the strong FLP in Ti‐contacted devices limits the top gate‐tunability of *R*_jun_, resulting in unchanged *R*_C_. TCAD simulations provided further confirmation of the contribution of *V*_TG_ to *R*_jun_ and the overall *R*_C_ improvement of Sb‐contacted MoS_2_ devices. The FLP‐free Sb contact in DG device architecture has a significant advantage in the effective gate control of *R*_C_, which can help achieve high‐performance 2D FETs.

## 4 Experimental Section

### Device Fabrication Process

The 2D materials (hBN and MoS_2_) were mechanically exfoliated using the scotch‐tape method before being transferred onto a degenerately p‐doped Si wafer covered by 285 nm of thermally grown SiO_2_, which is served as a global gate dielectric. The thickness of MoS_2_ flakes (<5 nm) was identified by optical contrast. PMMA A6 resist was spin‐coated to form a mask, followed by the electron beam lithography (EBL) process for device patterning. Subsequently, Sb/Au (Ti/Au) metal contacts with a thickness of 5/20 (5/20) nm were directly deposited with an e‐beam evaporator at a vacuum pressure of 5 × 10^−7^ Torr. The top dielectric was formed by covering the whole structure with hBN flake (≈50 nm) via a dry pick‐up and transfer process. The top gate electrode was formed through EBL patterning followed by a Ti/Au (5/70) deposition process.

### Device Characterization

All electrical measurements were performed using a semiconductor parameter analyzer (Agilent 4155C) connected to a vacuum probe station with pressure maintained at 20 mTorr.

### TCAD Simulations

Device analysis was performed through Synopsys Sentaurus (Synopsys Inc., Mountain View, CA, USA), a 3D technology computer‐aided design (TCAD) software package. To describe the energy band characteristics of MoS_2_ as a channel, the room temperature effective density‐of‐states *N*_C_ (300 K) and *N*_V_ (300 K) of 7.947 × 10^19^ cm^−3^ and 9.674 × 10^19^ cm^−3^, respectively, the bandgap of 1.2 eV, and dielectric constants of *ε*_⊥_ = 6.4 and *ε*_∥_ = 15.1 were used.^[^ ^37^ ^]^ The work function of 4.4 eV was used for both Sb and Ti metals, and SBH = 0 eV was realized at the interface between MoS_2_ and Sb due to its FLP‐free nature. In addition, the Sze model among the FLP models was used to describe the interface characteristics of MoS_2_ and Ti. The density of interface states per unit energy (*N*_I_) of 8.5 × 10^12^ cm^−2^ eV^−1^, their extent into the semiconductor (*d*) of 2 × 10^−7^ cm, and charge neutrality level (*E*_CNL_) of 5.00 eV were used to implement Schottky barrier height of 0.2 eV.

## Conflict of Interest

The authors declare no conflict of interest.

## Supporting information

Supporting Information

## Data Availability

The data that support the findings of this study are available from the corresponding author upon reasonable request.
