# Supplementary material for: Modulation of Contact Resistance of Dual‐Gated MoS2 FETs Using Fermi‐Level Pinning‐Free Antimony Semi‐Metal Contacts
Source: Adv Sci (Weinh). 2023 May 5;10(21):2301400. doi: 10.1002/advs.202301400 (PMC10375162; doi:10.1002/advs.202301400)
Supplement: Supplementary file 1 — Supporting Information [file ADVS-10-2301400-s001.pdf]

ADVANCED SCIENCE
Open Access

Supporting Information

for *Adv. Sci.*, DOI 10.1002/advs.202301400

Modulation of Contact Resistance of Dual-Gated MoS_2 FETs Using Fermi-Level Pinning-Free Antimony Semi-Metal Contacts

Tien Dat Ngo, Tuyen Huynh, Hanggyo Jung, Fida Ali, Jongwook Jeon, Min Sup Choi and Won Jong Yoo**

Supporting Information

Modulation of Contact Resistance of Dual-Gated MoS_2 FETs Using Fermi-Level Pinning-Free Antimony Semi-Metal Contacts

Tien Dat Ngo[a], Tuyen Huynh[a], Hanggyo Jung[b], Fida Ali[c], Jongwook Jeon[b], Min Sup Choi[d,*] and Won Jong Yoo [a,*]

[a]SKKU Advanced Institute of Nano Technology, Sungkyunkwan University, Suwon, Gyeonggi-do 16419, Republic of Korea

[b]Department of Electrical and Electronics Engineering, Konkuk University, Seoul 05029, Republic of Korea

[c]Department of Electronics and Nanoengineering, Aalto University, P.O. Box 13500, Aalto FI-00076, Finland

[d]Department of Materials Science and Engineering, Chungnam National University, Daejeon 34134, Republic of Korea

[*] e-mail: goodcms@cnu.ac.kr, yoowj@skku.edu

Raman and AFM characterizations of the 2D materials

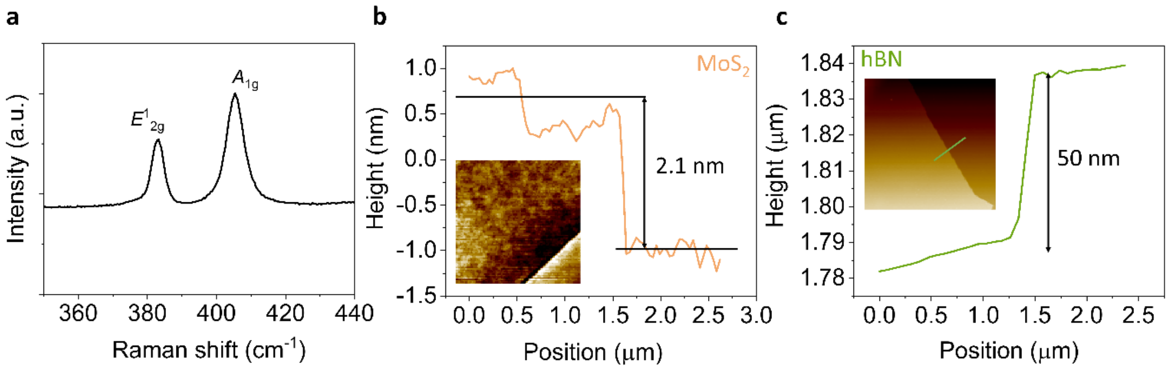

Figure S1. a) Raman spectrum of MoS_2 flake. Thickness and topography of b) MoS_2 flake and c) hBN measured by AFM.

Electrical performance of Sb-contacted MoS_2 Dual-gated (DG) device

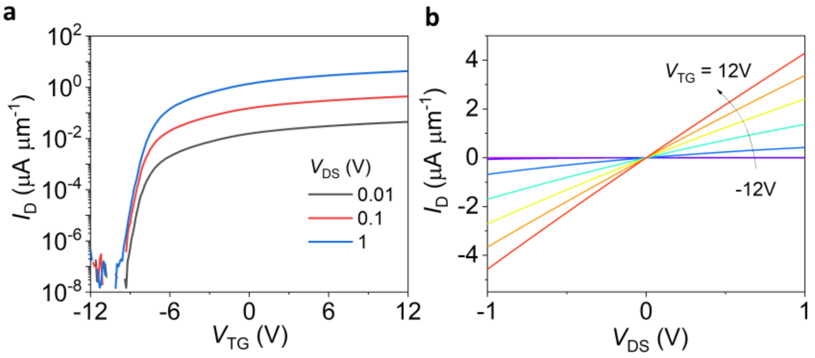

Figure S2. a) Top-gate transfer characteristics of the Sb-contacted DG FET at $V_{BG} = 0$ V. b) Output characteristic of the FET by varying V_{TG} = -12 to 12 V (at $V_{BG} = 0$ V).

Figure S2 represents the electrical performance of Sb-contacted device by varying V_{TG} from -12 to 12 V at $V_{BG} = 0$ V. Without V_{BG}, Ohmic behavior still can be obtained for Sb-contacted device, which further demonstrates the superiority of Sb semimetal over normal metals for 2D MoS_2 FETs.

Mobility and hysteresis of Sb-contacted MoS_2 DG FETs

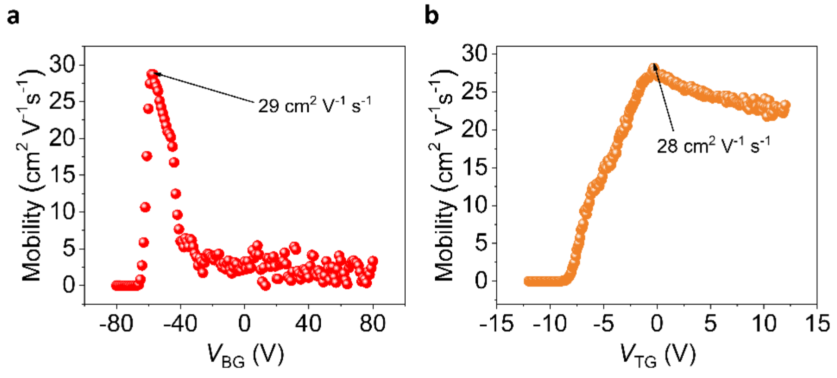

Figure S3. Mobility of Sb-contacted device as a function of a) V_{BG} and b) V_{TG}.

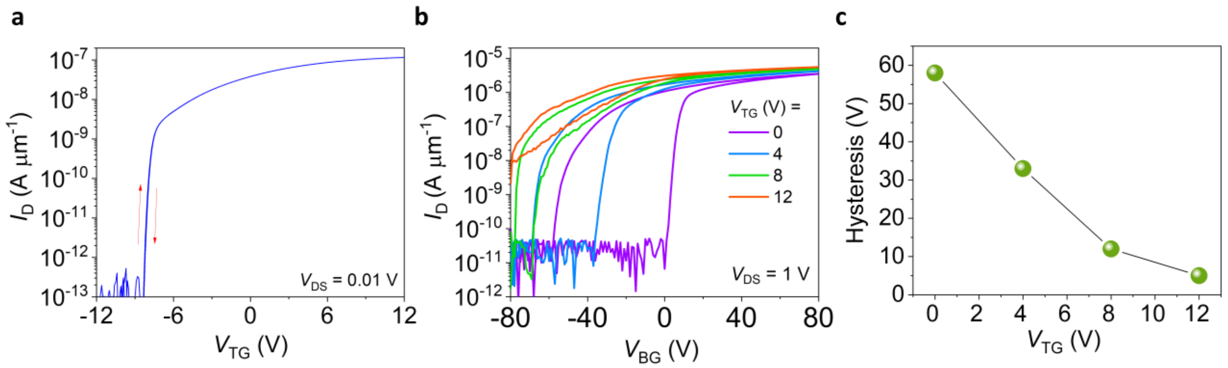

Figure S4. Double sweep transfer characteristics a) I_D-V_{TG} and b) I_D-V_{BG} with varied V_{TG}, and c) hysteresis window as a function of V_{TG} for Sb-contacted device.

The mobilities of Sb-contacted device with V_{BG} and V_{TG} are shown in **Figure S3** (we used the extraction method of field-effect mobility referring to Fuhrer et al. *Nat. Nanotechnol.* **2013**, 8, 146). The mobilities as a function of both V_{TG} and V_{BG} showed a similar peak mobility. However, the saturated mobilities at high V_{TG} showed higher values compared to those at high V_{BG} presumably due to charge trapping at MoS_2/SiO_2 interface as we used SiO_2 as global back-gate in this work. The charge trapping also can be evidenced by hysteresis as shown in **Figure S4**. Interestingly, we found that the hysteresis is significantly suppressed by applying V_{TG} as shown in

Figure S4b and c, which can be another merit of DG structure. Despite the hysteresis in transfer curves, our main finding and interpretation on the effect of FLP-free contacts in DG FETs are still valid as we used the same SiO_2 substrate for both Sb and Ti metal contacts and I_D-V_{TG} did not reveal a significant hysteresis as shown in Figure S4a.

Electrical performance of a Ti-contacted MoS_2 DG device

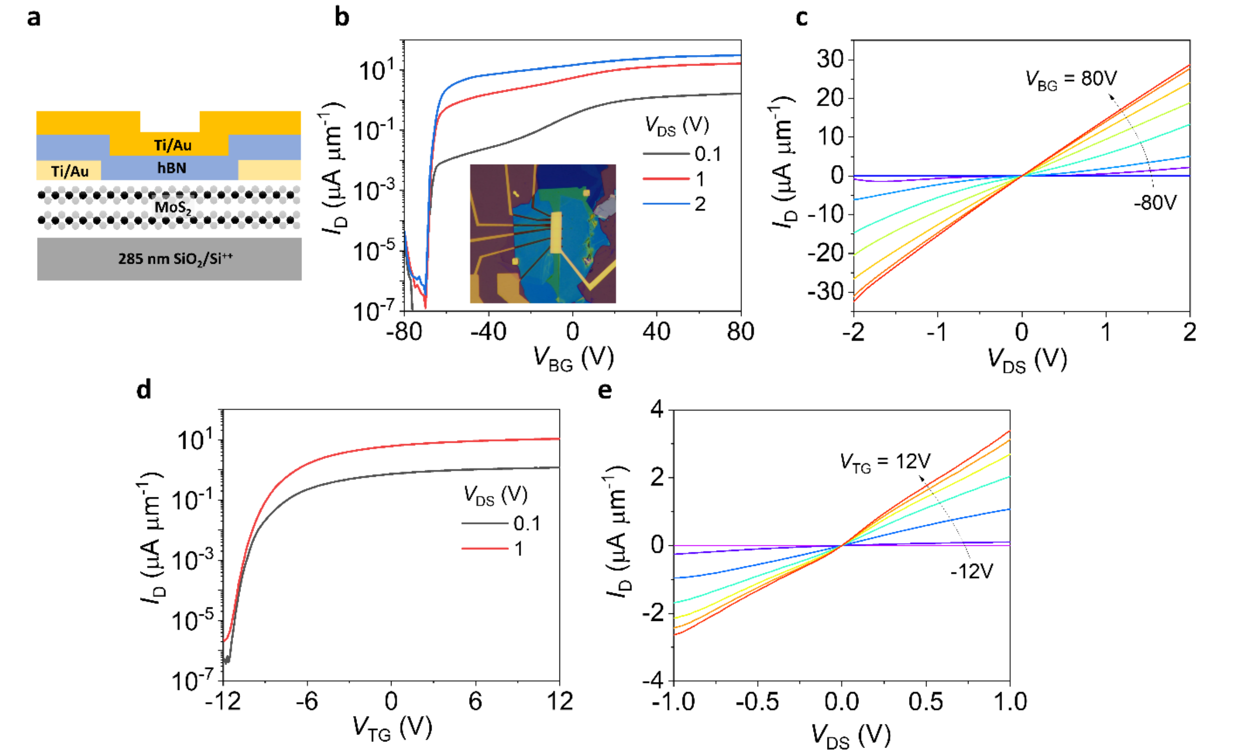

Figure S5. a) Schematic of a Ti-contacted DG MoS_2 FET. b) Transfer characteristics and c) output characteristics of the device at V_{TG} = 0 V. The inset in (b) shows an optical image of the device. d) Top-gate transfer characteristics of the Ti-contacted DG FET at V_{BG} = 0 V. e) Output characteristic of the FET by varying V_{TG} from -12 to 12 V (at V_{BG} = 0 V).

Figure S5 shows electrical performances of a Ti-based DG MoS_2 FET obtained at V_{TG} = 0 V. Figure S5b represents transfer curves of the device (L_{ch} = 7.8 µm) as a function of V_{BG} with

varied V_{DS}. A non-linear and asymmetric behavior is observed with small V_{BG} and V_{TG} in the Ti-based devices, indicating Fermi level pinning (FLP) behavior with the normal metal (Figures S5c,e).

Electrical performance of DG devices with varying V_{TG}

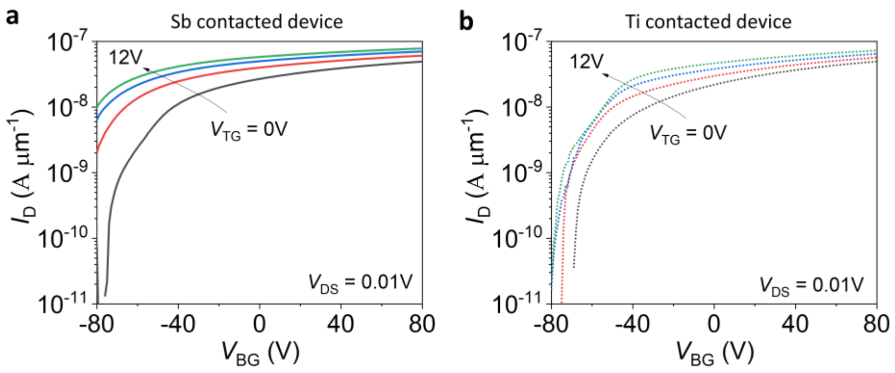

Figure S6. a) Transfer characteristics of the a) Sb- and b) Ti- contacted FETs with V_{DS} = 0.01 V and varied V_{TG} = -12 to 12 V.

Figure S6 represents the transfer characteristics of Sb- and Ti-contacted DG FETs with various V_{TG}. Both devices show a similar improvement in on-state current when V_{TG} increases. However, the threshold voltage (V_{th}) of Sb-contacted device is strongly shifted to negative voltage, while Ti-contacted device revealed a slight V_{th} shift. The origin of such different transfer characteristics can be evidenced from R_{sh} and R_C tunability as a function of carrier densities in these devices, as shown in **Figure 3**a,b in the main text. R_{sh} of both devices demonstrate a similar trend and values while R_C tunability is clearly different for both contacts. Thus, we think that the difference in transfer characteristics of Sb- and Ti-contacted DG FETs with various V_{TG} is mostly originated from strong V_{TG} dependent R_C of Sb-contacted device compared to weak V_{TG} dependent R_C of Ti-based device.

Extraction of Schottky barrier heights (SBHs) of Sb- and Ti-contacted DG MoS_2 devices from temperature dependent transfer characteristics

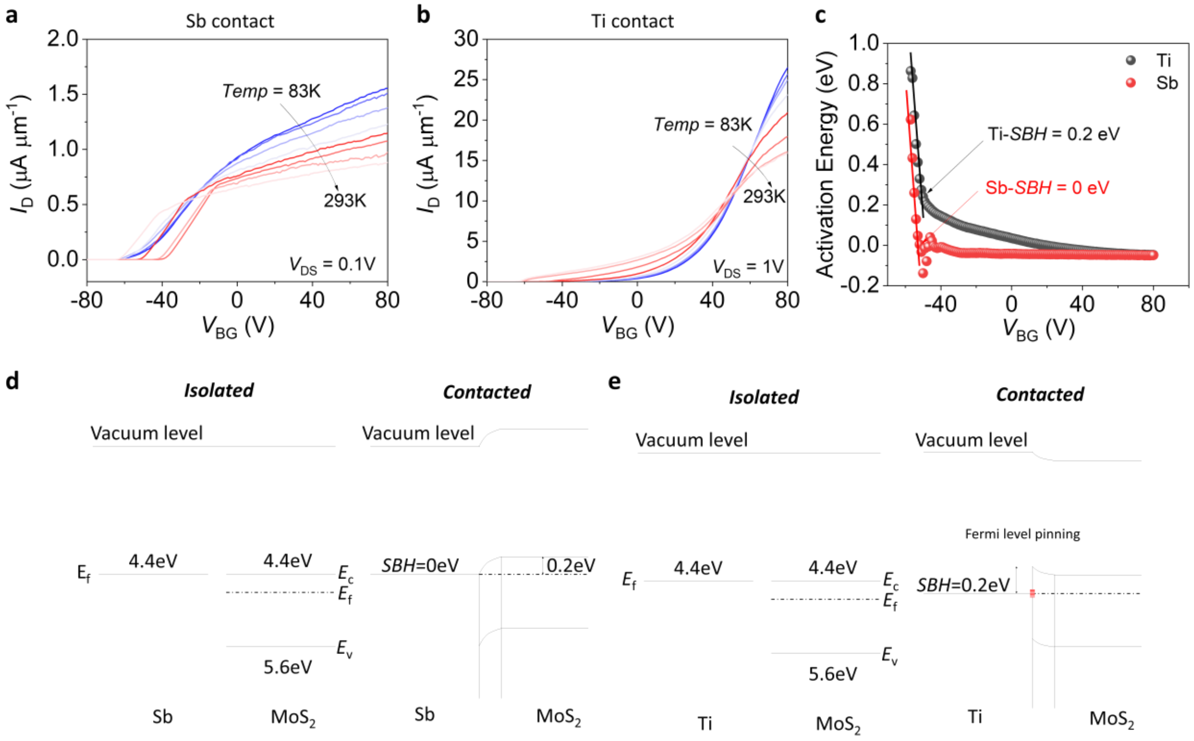

Figure S7. Temperature dependent transfer characteristics of a) Sb- and b) Ti-contacted DG MoS_2 devices (V_{TG} = 0V). c) SBHs of the devices with both metallic contacts obtained from Arrhenius plots. Band diagrams of d) Sb- and e) Ti-contacted DG MoS_2 devices at isolated and contacted states.

For 2D Schottky contacted FETs, which can be considered as two back-to-back diodes, the SBHs can be extracted by the following thermionic emission equation:

$$I_{2D} = WA_{2D}^{*}T^{3/2}\exp(-\frac{q\phi_{Bn}}{kT})\exp(\frac{qV_D}{kT}), A_{2D}^{*} = \frac{q\sqrt{8\pi m^{*}k^{3}}}{h^{2}}, \quad (1)$$

$$\phi_{Bn} = \frac{k}{q}\left[-\frac{\Delta\ln(I_D/T^{3/2})}{\Delta T^{-1}}\right]. \quad (2)$$

To obtain Arrhenius relationship between drain current and temperature, we measured temperature dependent transfer characteristics of the Sb- and Ti-contacted devices, as shown in **Figure S7**a and b. By using equations (1) and (2), we extracted SBHs of the devices (Figure S7c). The extracted SBHs are 0.2 and 0 eV for Ti- and Sb-contacted devices, respectively. Based on the extracted SBHs, we expected the band alignment of the device at isolated state and contacted states as depicted in Figure S7d and e. For the Sb-based device, the negligible SBH indicates no difference in Fermi levels between Sb and MoS_2 due to Fermi level de-pinning effect. However, for the Ti-contacted device, a considerable SBH of 200 meV is formed unlike the expected band alignment at isolated state due to strong FLP with conventional metallic contacts. Thus, we believe that the distinct V_{TG} dependence of Sb-contacted device originates from the different nature of the contacts.

Sb-contacted DG MoS_2 FETs prepared separately with the device in the main text

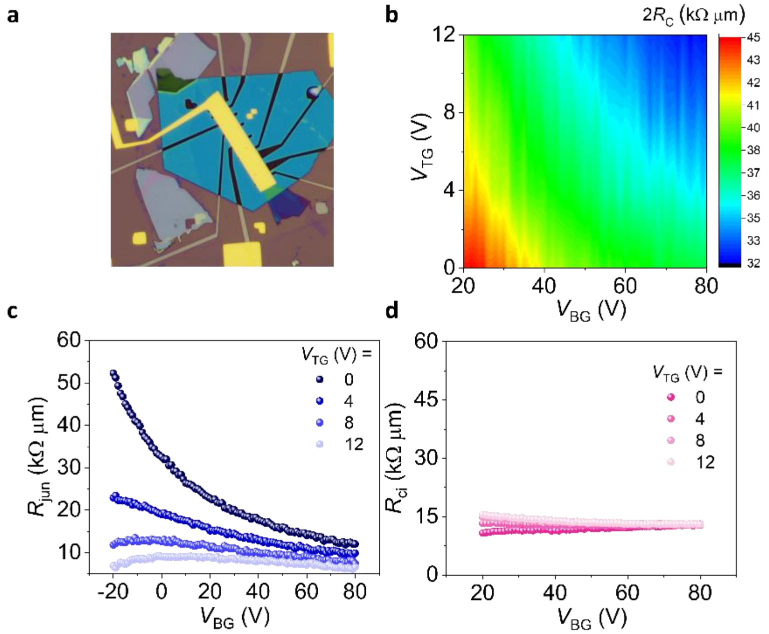

Figure S8. a) Optical image of an Sb-contacted DG MoS_2 FET (2nd device). b) $2R_C$ map, c) R_{jun}, and d) R_{ci} as a function of V_{TG} and V_{BG} for the device.

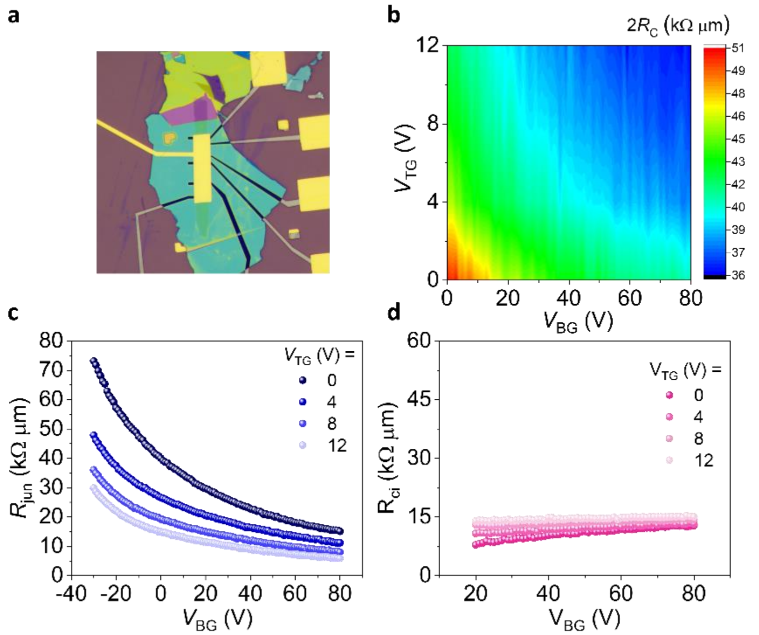

Figure S9. a) Optical image of an Sb-contacted DG MoS_2 FET (3rd device). b) $2R_C$ map, c) R_{jun}, and d) R_{ci} as a function of V_{TG} and V_{BG} for the device.

4PP measurements of the Ti-contacted DG MoS_2 FET

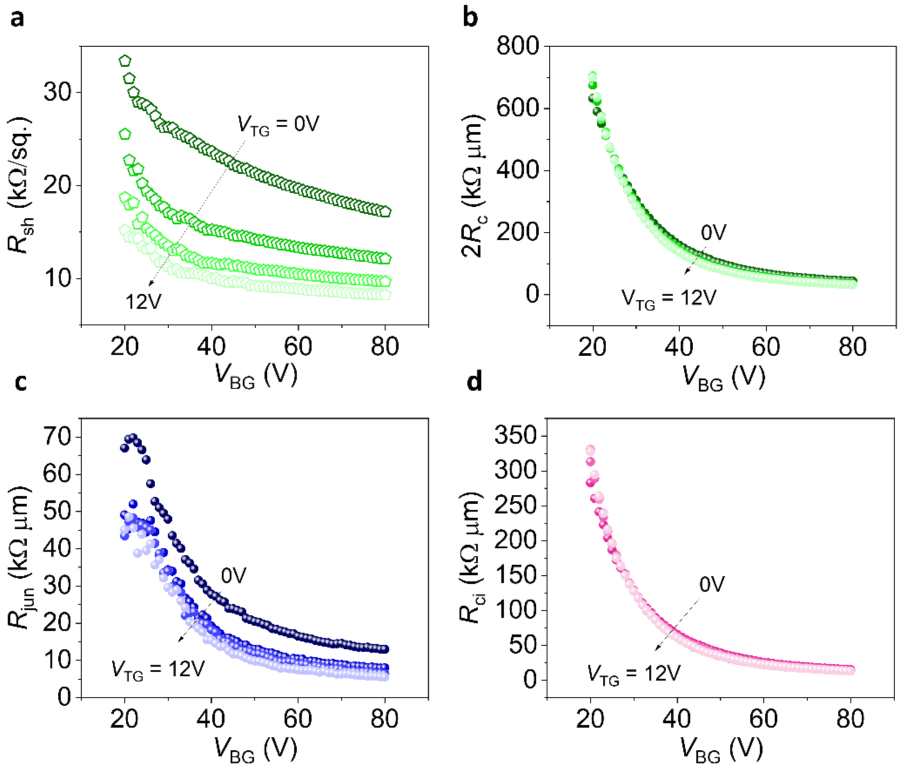

Figure S10. a) R_{sh}, b) $2R_C$, c) R_{jun}, and d) R_{ci} as a function of V_{BG} at different V_{TG} for Ti-contacted DG MoS_2 FET.

Figure S10a demonstrates that R_{sh} is effectively tuned by V_{TG} as the MoS_2 channel is exposed to V_{TG}-induced electric field (E_{TG}). However, the R_C including R_{jun} and R_{ci} remains almost unchanged by V_{TG} as shown in Figure S10b-d. The weakly modulated R_{jun} by V_{TG} is attributed to the strong FLP in the Ti-contacted device, which is further confirmed by TCAD simulation in **Figure S11**. It is noted that the R_{ci} is also unaffected by V_{TG} due to screening effect of the top-gate metal electrode.

TCAD simulations for contact resistance components of Ti-MoS_2 DG FETs

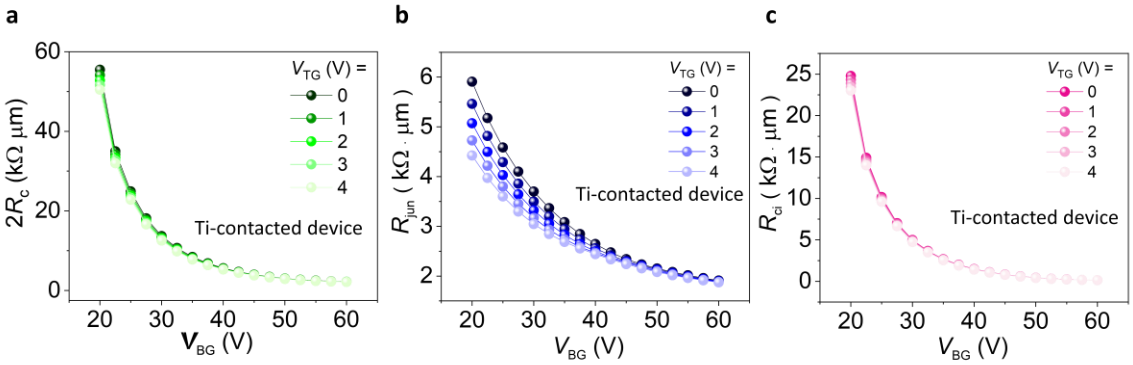

Figure S11. TCAD simulations for b) 2R_C, c) R_{jun}, and d) R_{ci} as a function of V_{BG} and V_{TG} for the Ti-contacted DG MoS_2 FET.

Table S1. Parameters for TCAD simulations.

**Parameters**	**Values**
**Dimension**	
Channel length	11.5 μm
Channel thickness	5 nm
S/D length	1 μm
hBN thickness	50 nm
SiO_2 thickness	285 nm
**Channel (MoS_2)**	
Effective density-of-states in conduction band at 300K	7.947×10^{19} cm^{-3}
Effective density-of-states in valence band at 300K	9.674×10^{19} cm^{-3}
Energy band gap	1.2 eV
Dielectric constant of in-plane	6.4
Dielectric constant of Out-of-plane	15.1
Channel dopant doping	1×10^{17} cm^{-3}
**Metal (type : Sb) – Fermi level pinning-Free**	
Transfer length	7 nm
Contact resistivity	0.225 kΩ · $μm^2$
Doping under S/D	5×10^{18} cm^{-3}
Lateral doping	2×10^{18} cm^{-3}
Lateral junction length	10 nm
Schottky barrier height w/ channel	0.0 eV
**Metal (type : Ti) – Strong Fermi-level pinning**	
Transfer length	150 nm
Contact resistivity	0.225 kΩ · $μm^2$
Density of interface states per unit energy	8.5×10^{12} cm^{-2} · eV^{-1}
Extent into the semiconductor	2×10^{-7} cm
Charge neutrality level	5.00 eV
Schottky barrier height w/ channel	0.2 eV

TCAD simulations for contact resistance components of Sb-MoS_2 DG FETs with varied transfer lengths and junction lengths

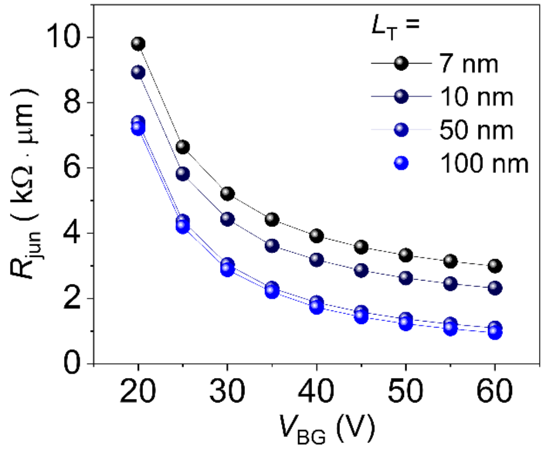

Figure S12. TCAD simulation for R_{jun} as a function of V_{BG} with varied transfer lengths (L_T).

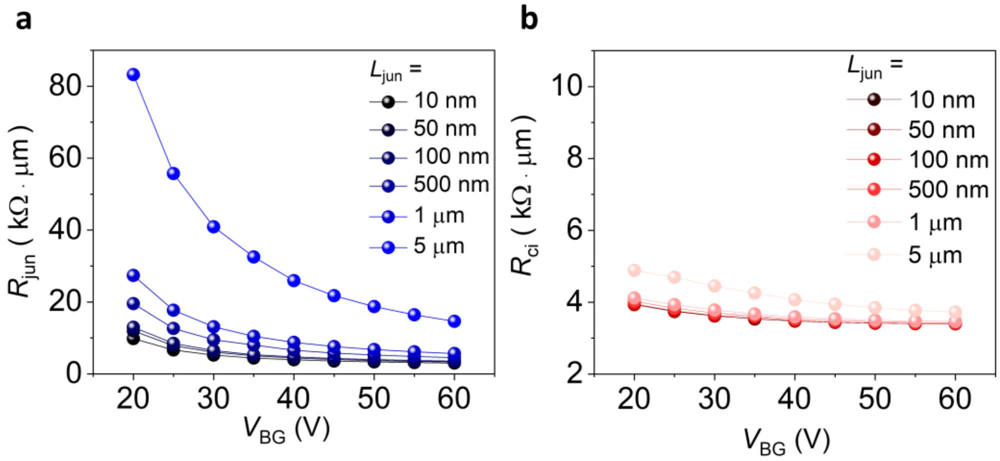

Figure S13. TCAD simulation for a) R_{jun} and b) R_{ci} as a function of V_{BG} with varied junction lengths (L_{jun}).
